# COVID-19 associated Mucormycosis (CAM): Should Brazil be on alert?

**DOI:** 10.1590/0037-8682-0410-2021

**Published:** 2021-09-06

**Authors:** Luís Arthur Brasil Gadelha Farias, Lisandra Serra Damasceno, Silviane Praciano Bandeira, Francisca Kalline de Almeida Barreto, Terezinha do Menino Jesus Silva Leitão, Luciano Pamplona de Góes Cavalcanti

**Affiliations:** 1 Escola de Saúde Pública do Ceará, Fortaleza, CE, Brasil.; 2 Hospital São José de Doenças Infecciosas, Fortaleza, CE, Brasil.; 3 Universidade Federal do Ceará, Faculdade de Medicina, Departamento de Saúde Comunitária, Fortaleza, CE, Brasil.; 4 Universidade Federal do Ceará, Faculdade de Medicina, Departamento de Patologia e Medicina Legal, Fortaleza, CE, Brasil.; 5 Centro Universitário Christus, Faculdade de Medicina, Fortaleza, CE, Brasil.

Dear Editor:

Mucormycosis is an emerging fungal infection that induces a life-threatening disease caused by zygomycetes belonging to the order *Mucorales.* There are several genera in this group of fungi. The most important are the *Rhizopus* sp., *Mucor* sp., *Rhizomucor* sp., *Syncephalastrum* sp., and *Lichtheimia* sp. The zygomycetes group was identified based on morphological characteristics including fungal hyphae typical for mucormycetes in biopsies of affected tissues or bronchoalveolar lavage, direct microscopy on KOH, and culture identification with antifungal susceptibility test. However, identification at the species level is difficult^1^ ^-^ ^4^. Molecular-based methods may be applied to tissues for species identification. Other method such as matrix-assisted laser desorption ionization-time of flight mass spectrometry for the identification of cultured *Mucorales* is promising; however, more validated data are needed. Thus, molecular identification remains the gold standard^3^ ^,^ ^4^.

**FIGURE 1:**
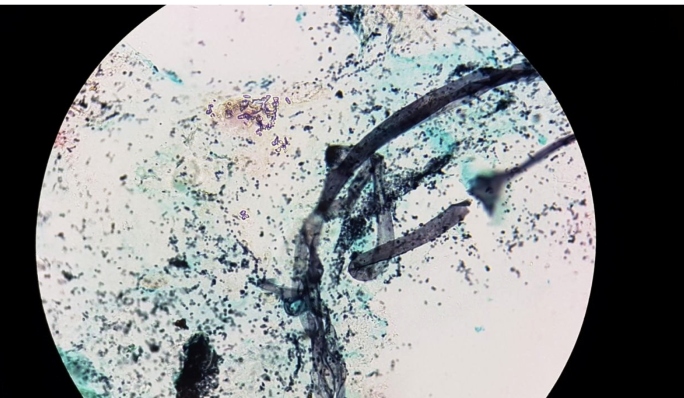
Broad and coenocytic hyphae in paranasal sinus content biopsy (Gomori methenamine silver - 1000x).

The zygomycetes are hyaline filamentous fungi with coenocytic and broad hyphae with branches oriented at 90°, and septations are rarely observed (Figure 1). In the culture media, they are characterized by grayish colonies with abundant mycelia. These saprophytic microorganisms thrive in soil, decaying organic matter, fruits, and starchy foods. Inhalation of fungal spores results in the colonization of the human airways^1^ ^,^ ^3^ ^,^ ^4^.

Brazil is responsible for most published cases of mucormycosis in Latin America (Brazil, 59; Argentina, 36; Chile, 14; Colombia, 22; Venezuela, 7; Peru, 3; and Ecuador and French Guiana, 1 case each) as identified by Nucci M. et al^5^. Patients with mucormycosis from Brazil were predominantly male (n=37; 62.5%) with a median age of 43 (<1-78) years. The most common underlying conditions of the affected individuals include diabetes mellitus (42.4%), solid organ transplant (22.9%), and malignancy (16.9%). In Brazil, the mortality rate of mucormycosis was higher (52.5%) than overall mortality due to other reasons (48.3%); it was also higher than the mortality rates of mucormycosis reported from other countries (45.2%).The identified genera from Brazil included *Rhizopus* sp. (n=11; 18.6%), *Mucor* sp. (n=7; 11.9%), and rarely *Rhizomucor* sp. (n=2; 3.5%), and *Lichtheimia* sp. (n=2; 3.5%)^5^. 

Although mucormycosis is a rare invasive fungal infection, its emergence has increased during the coronavirus disease-2019 (COVID-19) pandemic^1^ ^,^ ^2^. In particular, the occurrence of fungal infections, such as *Candida sp.* and *Aspergillus sp.* co-infections has increased during the pandemic^2^ ^,^ ^6^. The burden of mucormycosis in India emphasizes the high incidence of lethal fungal infections and its implications for public health^7^ ^-^ ^10^. 

According to the World Health Organization (WHO), 28,307,832 COVID-19 cases and 335,102 deaths from COVID-19 were reported in India in June 2021^7^. The emergence of COVID-19-associated mucormycosis (CAM) is not coincidental. The main risk factors may be the high incidence of diabetes in the country's population (many without diagnosis and treatment), favoring the outbreak of mucormycosis in hospitalized intensive care unit patients receiving high-dose corticosteroids for COVID-19 treatment^1^ ^,^ ^2^.

Mucormycosis outbreaks in India were initially reported by ophthalmology professionals during the COVID-19 pandemic^8^. As a part of COVID-19 treatment, diabetes patients received increased corticosteroids, leading to uncontrolled glucose levels, resulting in a fertile ground for the *Mucorales* fungi. John et al. identified 43 cases of mucormycosis in COVID-19 patients and found that 33 of 35 (94%) patients had documented glycemic status representative of diabetes^9^. In a recent retrospective study by Patel et al., uncontrolled diabetes was the most common underlying disease among COVID-19 patients admitted from September 2020 to December 2020, including 187 patients with CAM. Other factors included hematologic malignancy and SOT^10^. 

Diabetes increases the risk of worse outcomes in patients infected with severe acute respiratory syndrome-coronavirus-2^11^ ^-^ ^13^. Thus, the need for hospitalization has increased due to the high number of patients with diabetes in Brazil^13^. The prevalence of diabetes is approximately 11.5%, and most patients are between 20 and 69 years of age. Moreover, these patients commonly have uncontrolled hyperglycemia^12^. In a study conducted in Brazil, diabetes patients with COVID-19 were more frequently admitted to the intensive care units (18%). Specifically, they were 1.34 times more likely to require hospitalization (1.10-1.61, p=0.003)^13^. Furthermore, the hyperinflammation generated by COVID-19 makes glycemic control difficult. There has been one reported death attributed to mucormycosis in a 56-year-old man with diabetic ketoacidosis from Manaus, Amazonas, located in northern Brazil^14^.

Although mucormycosis has been prevalent in India (71% of global cases), some cases have also been reported in Brazil^14^ ^,^ ^15^. According to PAHO/WHO, seven American (USA, Mexico, Brazil, Chile, Honduras, Paraguay, and Uruguay) countries have reported confirmed CAM cases^15^. 

To standardize the surveillance and control of invasive fungal infections related to COVID-19, Anvisa published a technical note (GVIMS/GGTES/ANVISA Nº 04/2021) with the diagnostic criteria for suspected mucormycosis. The criteria included decompensated diabetes patients diagnosed with severe COVID-19, who were administered steroids during or after COVID-19 and had acute/subacute sinusitis, with imaging documentation of sinusitis and at least one of the following signs: (1) early: acute and localized (including pain radiating to the eye) pain, fever, impaired general condition, and severe facial pain; or (2) delayed: nasal ulcer with black exudate, nasal bleeding, facial edema, asymmetries, eye pain, eyelid ptosis, visual changes, amaurosis, freezing of eye movements, and necrosis around the nose. Mycosis can extend to the paranasal sinus and bone barriers, including the orbit and the palate, as well as affecting the central nervous system via abscess formation^16^. Other differential diagnoses that may involve progressive facial swelling, ulceration, and destruction must be excluded. Furthermore, mucormycosis may resemble orbital cellulitis, extranodal T-cell lymphoma, or cutaneous anthrax^1^. 

With the emergence of new variants, it is crucial to prepare the public health system structurally and professionally, considering the repercussions of zygomycete infections in India. The delta COVID-19 variant (B.1.617.2), first detected in India, has spread to other countries, such as the United Kingdom, and poses the risk of a new COVID-19 surge^14^. The emergence of new variants in Brazil has led to concerns among the health professional and scientific community regarding potential complications, including the impact of fungal infections on COVID-19 morbidity and mortality^1^ ^,^ ^8^ ^,^ ^17^.

Hospitals should be prepared to diagnose and treat serious fungal infections requiring surgery and make available expensive antifungal agents. Currently, treatment is based on a multimodal approach, including the control of underlying predisposing factors, early administration of active antifungal agents at optimal doses, and complete removal of any infected tissues. Ampotericin B lipid-based formulations formulations are the antifungal therapy of choice^1^ ^,^ ^6^ ^,^ ^14^ ^,^ ^16^. Other antifungal therapies such as posaconazole and isavuconazole are recommended alternatives^16^. Nonetheless, the health systems must be aware of and prepared to treat invasive fungal infections in the COVID-19 era.
